# The Susceptive Alendronate-Treatment Timing and Dosage for Osteogenesis Enhancement in Human Bone Marrow-Derived Stem Cells

**DOI:** 10.1371/journal.pone.0105705

**Published:** 2014-08-26

**Authors:** Chih-Hsiang Chang, Chau-Zen Wang, Je-Ken Chang, Che-Yu Hsu, Mei-Ling Ho

**Affiliations:** 1 Orthopaedic Research Center, Kaohsiung Medical University, Kaohsiung, Taiwan; 2 Department of Physiology, College of Medicine, Kaohsiung Medical University, Kaohsiung, Taiwan; 3 Department of Orthopaedics, College of Medicine, Kaohsiung Medical University Hospital, Kaohsiung Medical University, Kaohsiung, Taiwan; 4 Department of Orthopaedics, Kaohsiung Municipal Ta-Tung Hospital, Kaohsiung, Taiwan; Rutgers - New Jersey Medical School, United States of America

## Abstract

Recent studies indicated that alendronate enhanced osteogenesis in osteoblasts and human bone marrow-derived stem cells. However, the time- and dose-dependent effects of Aln on ostegenic differentiation and cytotoxicity of hBMSCs remain undefined. In present study, we investigated the effective dose range and timing of hBMSCs. hBMSCs were treated with various Aln doses (1, 5 and 10 µM) according to the following groups: group A was treated with Aln during the first five days of bone medium, groups B, C and D were treated during the first, second, and final five days of osteo-induction medium and group E was treated throughout the entire experiment. The mineralization level and cytotoxicity were measured by quantified Alizarin Red S staining and MTT assay. In addition, the reversal effects of farnesyl pyrophosphate and geranylgeranyl pyrophosphate replenishment in group B were also investigated. The results showed that Aln treatment in groups A, B and E enhanced hBMSC mineralization in a dose-dependent manner, and the most pronounced effects were observed in groups B and E. The higher dose of Aln simultaneously enhanced mineralization and caused cytotoxicity in groups B, C and E. Replenishment of FPP or GGPP resulted in partial or complete reverse of the Aln-induced mineralization respectively. Furthermore, the addition of FPP or GGPP also eliminated the Aln-induced cytotoxicity. We demonstrated that hBMSCs are susceptible to 5 µM Aln during the initiation stage of osteogenic differentiation and that a 10 µM dose is cytotoxic.

## Introduction

Bisphosphonates are clinically used to inhibit osteoclast activity and to treat osteoporosis. Alendronate (Aln), an aminobisphosphonate, is used as an antiosteoporotic agent and this agent down-regulates osteoclast activity by inhibiting farnesyl pyrophosphate synthase (FPP synthase), thus blocking prenylation of guanosine triphosphate (GTP)-binding proteins [1], [2], [3], [4]. Moreover, Aln was also found to enhance osteogenic activities, including cell proliferation and osteo-differentation. The osteogenic effects of Aln were suggested due to increased alkaline phosphatase (ALP) activity, mineralization and osteogenic genes, such as bone morphogenetic protein-2 (BMP-2), type I collagen (Col I) and osteocalcin (OC) in osteoblasts [5], [6], [7], [8], bone marrow-derived stem cells (BMSCs) [9], [10] and human adipocyte-derived stem cells (human ADSCs) [11]. Previous studies suggested that Aln is uptaken by endocytosis of macrophages, osteoclasts and osteoblasts, the similar way may also happen in hBMSCs [12], [13].

A wide range of effective osteo-inducing Aln doses has been reported for different cell types. In MG-63 osteoblastic cells, 1 nM to 100 µM Aln promoted cell proliferation and maturation [7], [8]. In human BMSCs, 10 nM Aln enhanced osteo-differentiation [9], [10]; however, the enhancement of osteo-differentiation required more than 14 days of treatment [9], [10]. In our previous study, 5 µM Aln sufficiently enhanced osteo-differentiation in human ADSCs [11]. In contrast, aminobisphosphonate concentrations greater than 10 µM exerted strong inhibitory effects on both osteoblast proliferation and bone formation [6], [14]. Moreover, the Aln treatment periods for stem cells are typically less than four days [5], [7], [8], [9] or five days in osteo-induction medium [11]. The effective dose, treatment time and duration of Aln required for enhancing osteogenesis among different cell types were varied and those in mesenchymal stem cells have not been well characterized. Accordingly, in this study, we identified the appropriate treatment times and doses required for Aln-induced osteogenesis in human BMSCs. Simultaneously, the cell cytotoxic effects of Aln treatment were also investigated.

## Materials and Methods

### Reagents

Alendronate was obtained from Merck Pharmaceuticals (Whitehouse Station, NJ, USA). Alizarin Red S, thiazolyl blue tetrazolium bromide (MTT), Percoll, L-ascorbic acid-2-phosphate, beta-glycerolphosphate, sodium bicarbonate, dexamethasone, N-acetyl-L-cysteine (NAC), farnesyl pyrophosphate (FPP) and geranylgeranyl pyrophosphate (GGPP) were purchased from Sigma-Aldrich (Saint Louis, MO, USA). Dulbecco's Modified Eagle's Medium (DMEM), ascorbic acid, non-essential amino acids, penicillin/streptomycin, fetal bovine serum (FBS), keratinocyte-serum free medium (SFM), epidermal growth factor-bovine pituitary factor (EGF-BPE) and trypsin/EDTA were purchased from Gibco-BRL (Rockville, MD, USA). Dimethyl sulfoxide (DMSO) was purchased from Amresco LLC (Solon, OH, USA).

### hBMSC isolation and culture

Human bone marrow mesenchymal stem cells (hBMSCs) were isolated from bone marrow aspirated from iliac crest of three male donors with informed consent and approval from the hospital ethics committee (IRB number: KMUK-IRB-970267). The donors were aged 18 to 22 years without any known bone disorders and underwent surgery due to hip trauma. The nucleated stromal cells were separated from bone marrow fluid using a 70% Percoll gradient. Then, these cells were selected by K-NAC medium containing keratinocyte-SFM supplemented with the EGF-BPE, 5% fetal bovine serum, 2 mM N-acetyl-L-cysteine, 0.1 mM L-ascorbic acid-2-phosphate, and 0.5% penicillin (10000 U/ml)/streptomycin (10000 µg/ml) in a humidified atmosphere of 5% CO_2_ at 37°C. The hBMSCs were isolated in the K-NAC medium for 14 days after selection and the medium was changed every 3 days. The hBMSCs were used for experiments within 15 passages and the average doubling time of hBMSCs was 36–38 h. The hBMSCs were identified with CD markers as CD34−, CD45−, CD29+, CD49d+, CD90+ and CD166+ population [15], [16], and their multi-potent differentiation properties were confirmed by inducing to osteogenic, chondrogenic and adipogenic differentiation, which are shown in Figure S1.

### CD markers detection and multi-potent differentiation of isolated hBMSCs

Flow cytometry analysis was used to examine the presence of CD marker on the hBMSCs cell surface. Following the method of hBMSCs isolation, cells were harvested from the culture dishes by treating them with 0.25% trypsin/EDTA in PBS. One million hBMSCs were suspended in 500 µl of phosphate buffered saline (PBS) containing 20 µg/ml of an antibody. Phycoerythrin (PE)–conjugated antibodies (Becton Dickinson, Franklin Lakes, NJ) targeted against CD29, CD34, CD45, CD49d, CD90, CD166 were obtained from Becton Dickinson (BD; Becton Dickinson, Franklin Lakes, NJ). As an isotype control, PE-conjugated nonspecific mouse IgG was used as control. After incubation for 20 minutes at 4°C, the cells were washed with PBS 3 times and then suspended in 1 ml of PBS for analysis. Cell fluorescence was detected using a flow cytometer (FACS Vantage SE, Becton Dickinson) with a 570 nm filter for PE fluorescence, and the data were analyzed using WINMDI software.

We further analyzed the phenotypes of hBMSCs after induction into osteogenesis, chondrogenesis and adipogenesis. To induce osteogenic differentiation, cells were treated with OIM for 7 days and stained with ARS as described in methods. To induce chondrogenesis, cells were treated with chondro-induction medium (CIM) containing low glucose DMEM with 2.2 mg/ml sodium bicarbonate, 10% fetal bovine serum, 1% non-essential amino acid, 6.25 ug/mL insulin (Sigma-Aldrich, St. Louis, MO), 10 ng/mL TGF-β1 (Sigma-Aldrich, St. Louis, MO) 50 µmol/L L-ascorbate-2 -phosphate and 0.5% penicillin (10000 U/ml)/streptomycin (10000 µg/ml). After chondrogenic induction for 7 days, cells were fixed with 3.7% formaldehyde in phosphate buffered saline (PBS) for 30 minutes, washed once with PBS, and rinsed with distilled water; then they were processed for Alcian blue staining. Specimens were incubated with 0.05% Alcian blue solution overnight. Excess stain was removed by washing in PBS, rinsing with 5% acetic acid to remove nonspecific staining, and washing with PBS again. To induce adipogenesis, cells were treated with adipo-induction medium (AIM) containing Dulbecco's Modified Eagle's Medium supplemented with 0.5 mM 3-isobutyl-1-methylxanthine (Sigma-Aldrich), 1 µM dexamethasone (Sigma-Aldrich), and 10 µg/ml insulin. After 7 days, cells were washed twice with PBS and fixed with10% formalin in PBS for 1 hour; then washed three times with distilled water and dried. Cells were stained with oil red O for 15 minutes. Excess stain was removed by washing with 70% ethanol and distilled water.

### Osteogenic differentiation of hBMSCs and Aln treatment

**Figure 1:**
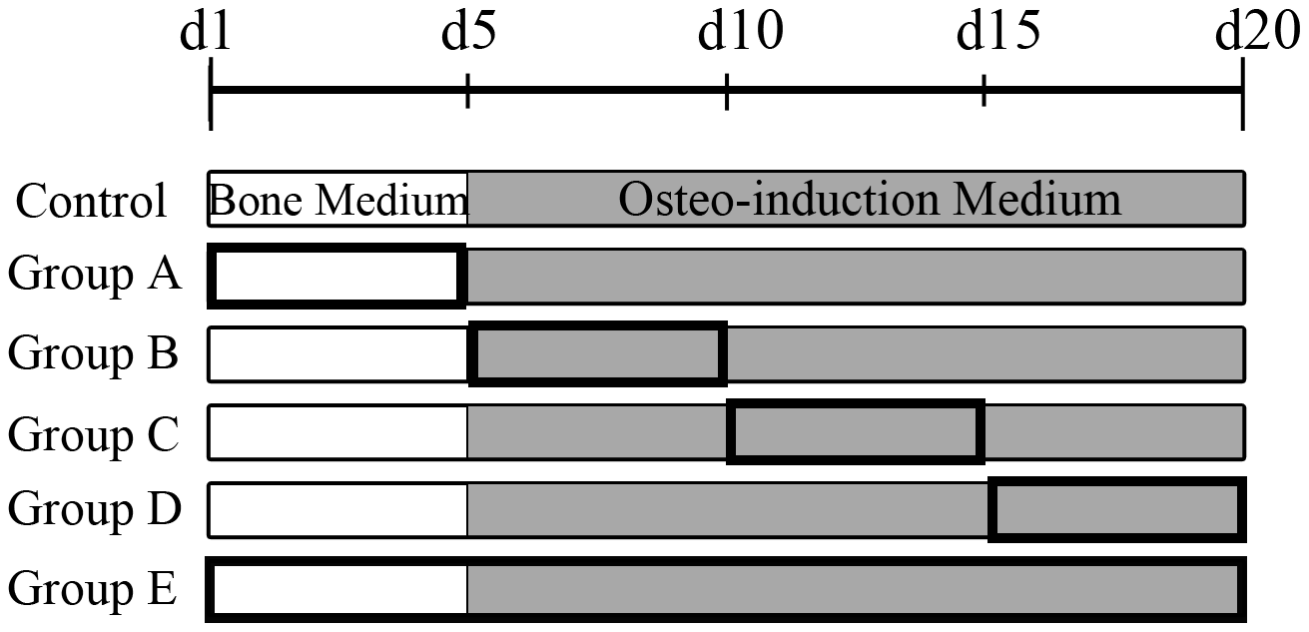
A schematic graph of Aln treatment strategies. All undifferentiated hBMSCs were seeded in 24-well plates and cultured for 5 days with bone medium (BM). Then, osteogenesis was induced using osteo-induction medium (OIM) for 15 days. The 5-day intervals of Aln treatments (indicated by bold frames) were performed during the first 5 days of culture (group A), day 6–10 (group B), 11–15 (group C), 16–20 (group D) or through entire experiment (group E). The control group was cultured with BM and then OIM without Aln treatment.

Isolated hBMSCs were seeded in 24-well plates at a density of 104 cells/well and cultured in bone medium (BM) containing low glucose DMEM with 2.2 mg/ml sodium bicarbonate, 10% fetal bovine serum, 1% non-essential amino acid solution, 1% ascorbic acid and 0.5% penicillin (10000 U/ml)/streptomycin (10000 µg/ml) in a humidified atmosphere of 5% CO2 at 37°C for 5 days. Osteogenesis was induced in the control group by culturing the cells for 20 days in the osteo-induction medium (OIM), which consisted of low glucose DMEM, 2.2 mg/ml sodium bicarbonate, 10% fetal bovine serum, 100 nM dexamethasone, 10 mM β-glycerophosphate, 0.1 mM L-ascorbic acid-2-phosphate, and 0.5% penicillin (10000 U/ml)/streptomycin (10000 µg/ml) after 5 days culture in BM. During the experimental period, the OIM was changed every 3 days. The hBMSCs were treated with various concentrations of Aln (1, 5 or 10 µM) at different culture stages, and the treatment groups are shown (Figure 1). In group A, cells were treated with Aln during the first five days (day 1–5) in BM. In group B, cells were treated with Aln during day 6–10 in OIM. In group C, cells were treated with Aln during day 11–15 in OIM. In group D, cells were treated with Aln during day 16–20 in OIM. In group E, cells were treated through entire experiment. In group B, 10 µM FPP or GGPP was replenished while 5 µM Aln treatment to test whether FPP or GGPP reverses Aln effect on mineralization.

### Alizarin Red S staining and quantitative analysis

Mineralization or extracellular matrix calcification in Aln-treated hBMSCs was estimated by Alizarin Red S (ARS) staining. On each indicated day, cultured cells were fixed with ice-cold 10% formalin for 15 minutes. After washing with distilled water, the cells were stained with 1% Alizarin Red S for 15 minutes. The stained cells were washed twice with distilled water and then quantified. The degree of mineralization was quantified by adding 400 µl 10% acetic acid per well at 60°C for 10 minutes to extract the Alizarin Red S staining. Then, 100 µl of supernatant was transferred to a 96-well plate, and the absorbance at 415 nm (OD415) was measured by spectrometry.

### MTT assay

The cell viabilities of Aln-treated hBMSCs were measured using MTT assay. On each indicated day, the cells were washed with PBS, and a one-tenth volume of MTT reagent (5 mg/ml stock) was added to each well, with a final volume of 500 µl in 24-well plate. The supernatant was carefully removed after 4 hours, and the formazan crystals were dissolved by adding 400 µl of DMSO per well at 37°C. The reactive product was transferred to an ELISA plate and the absorbance at 570 nm (with a reference absorbance in 655 nm) was measured by an ELISA reader (Tecan Sunrise, TECAN Deutschland GmbH, Crailsheim, Germany).

### RNA isolation and Real-time polymerase chain reaction (real-time PCR)

At indicated days, cells were collected from wells and extracted total RNA by TRIzol (Gibco BRL, Rockville, MD). Briefly, 0.5–1 µg of total RNA per 20 µl of reaction volume were reverse transcribed into cDNA using the SuperScript First-Strand Synthesis System (Invitrogen). Real-time PCR reactions were performed and monitored using the iQ SYBR Green Supermix (Bio-Rad Laboratories Inc, Hercules, CA) and quantitative real-time PCR detection system (iQ5, Bio-Rad Laboratories Inc, Hercules, CA). The cDNA samples (2 µl for total volume of 25 µl per reaction) were analyzed for genes of interest and the reference gene glyceraldehyde-3-phosphate-dehydrogenase (GAPDH). The relative mRNA expression levels were calculated from the threshold cycle (C_t_) value of each PCR product and normalized with GAPDH using the comparative C_t_ method [17]. All real-time PCR experiments were performed in triplicate and repeated at least three times. The primer sequences used were shown as followed: BMP-2: forward 5′-GGA ATG ACT GGA TTG TGG CT-3′; reverse 5′-TGA GTT CTG TCG GGA CAC AG-3′; Runx-2: forward 5′-AGA TGG GAC TGT GGT TAC TG-3′; reverse 5′-GTA GCT ACT TGG GGA GGA TT-3′; Osteocalcin: forward 5′-GTG CAG AGT CCA GCA AAG GT-3′; reverse 5′-CGA TAG GCC TCC TGA AAG C-3′; GAPDH: forward 5′-GAA GGT GAA GGT CGG AGT C-3′; reverse 5′-GAA GAT GGT GAT GGG ATT TC-3′.

### Statistical methods

All experiments were performed in triplicate. The data presented as means±standard deviation (SD) at a significance level of *p*<0.05. Statistical differences between groups were calculated using a one-way ANOVA, and multiple comparisons were performed by Scheffe's method.

## Results

### Alendronate enhanced hBMSC osteogenesis during the initiation stage of osteogenic differentiation

**Figure 2:**
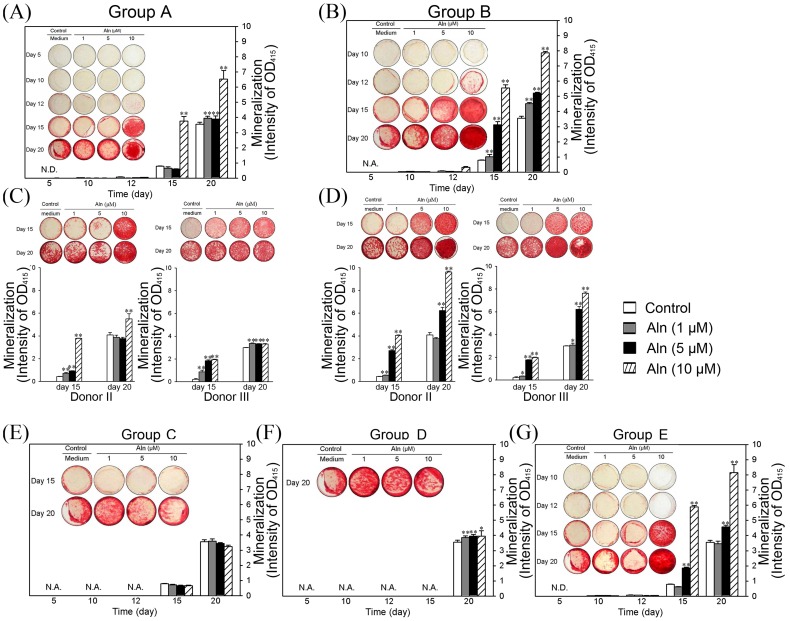
The mineralization effects of Aln on hBMSCs after different treatment strategies. Undifferentiated hBMSCs were treated with 1, 5 or 10 µM Aln during the culture time indicated in Figure 1. The mineralization levels were measured by ARS staining (shown as photographs), which was further quantified (shown as bar). The mineralization of group A (A) and group B (B) of donor I were measured at day 5, 10, 12, 15 and 20. The mineralization of donor II and donor III in group A (C) and group B (D) were measured at days 15 and 20. The mineralization of groups C (E), D (F) and E (G) of donor I were also determined using ARS staining (photographs) and quantified (bar). N.A: not available; N.D: not detectable; *p<0.05; **p<0.01 (n = 5).

The mineralization of induced hBMSCs were measured by ARS staining and quantified at the indicated days. As shown in Figure 2A, the ARS staining densities of group A were elevated with time. The quantified results showed that treatment with 10 µM Aln significantly increased mineralization after 15 days of treatment (*p*<0.01). The 1 µM or 5 µM Aln treatments did not significantly affect mineralization at day 15. At day 20, all doses of Aln significantly elevated mineralization, and 10 µM Aln showed the highest level of mineralization (Figure 2A, *p*<0.01). In group B, the mineralization levels were significantly elevated after Aln treatments in dose-dependent manner (Figure 2B). 1 µM and 5 µM Aln significantly elevated mineralization when compared to the control (Figure 2B, 1 and 5 µM, *p*<0.01), and 10 µM Aln treatment enhanced mineralization to a great degree than other doses at days 15 and 20 (Figure 2B, *p*<0.01). However, the mineralization levels of hBMSCs treated with Aln were not significantly different in group C (Figure 2E) and only weakly elevated in group D when compared to the control (Figure 2F). In group E, which consisted of hBMSCs treated with Aln during entire experiment, the mineralization effects were similar to those of group B (Figure 2G). The mineralization of hBMSCs treated with 5 µM Aln was significantly elevated at day 15 and 20 (Figure 2G, *p*<0.01), and hBMSCs treated with 10 µM Aln showed the highest degree of mineralization compared to the other doses (Figure 2G, *p*<0.01). In group A, gene expressions of BMP-2 and OC were significantly elevated in Aln treated hBMSCs than those in control group at day 5. Furthermore, the gene expressions of BMP-2, Runx-2 and OC in group B were significantly higher than those in group A and control group at day 7 (Figure S2A). We also measured the alkaline phosphatase (ALP) activity of control, group A and B at day 15. The result showed ALP activity in group B was the higher than other groups (Figure S2B).

We further confirmed the osteogenic effects observed in groups A and B on the hBMSCs obtained from donor II and III; the mineralization results were similar to those of donor I (Figure 2C and 2D). In group A of donor II and III, the osteogenic effects were significantly elevated after 10 µM Aln treatment at days 15 and 20 (Figure 2C, *p*<0.01). In group B, the elevated levels of mineralization were similar to those of donor I after treatment with 5 and 10 µM Aln, and the osteogenic effects were significantly increased at days 15 and 20 (Figure 2D, *p*<0.01). According to these results, hBMSCs were sensitive to the Aln-enhanced osteogenic effects during the initiation stage of incubation, regardless of the BM (group A) or OIM (group B) culture conditions. However, hBMSCs were insensitive to Aln in the later stage of OIM incubation, even after treatment with 10 µM Aln (groups C and D).

### High doses of Aln caused cytotoxicity

**Figure 3:**
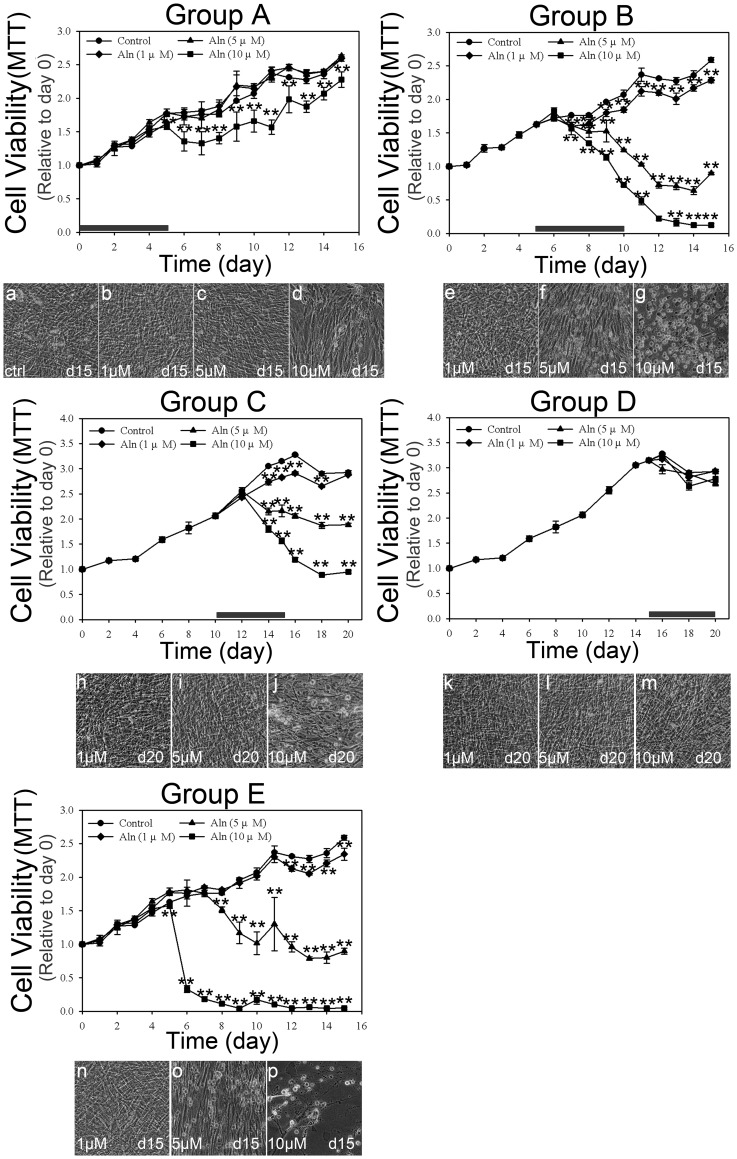
The cell viabilities of hBMSC after various Aln treatment strategies. The cell viabilities of cultures with various Aln treatment strategies were measured by MTT assays, and the cell morphology after various Aln doses for the indicated time are shown. The duration of Aln treatment is indicated by a bold bar. (Group A: a–d; Group B: e–g; Group C: h–j; Group D: k–m; Group E: n–p). **p<0.01 (n = 4).

The cell viabilities of Aln-treated hBMSCs in the five groups were also examined. As shown in Figure 3, cell viabilities were measured by MTT assay, and the cell morphology was observed by optical microscopy at the indicated times. Although MTT assay was associated to cell proliferation and mitochondrial activity, the reduction of MTT measurement indicated the loss of cell viability and also represented the cytotoxicity [18], [19]. In group A, hBMSCs treated with 1 or 5 µM Aln showed no difference when compared to the control cells; only treatment with 10 µM Aln significantly reduced the cell viability after day 6 (Figure 3, group A, *p*<0.01). At day 15, hBMSCs treated with 1 or 5 µM Aln were as confluent as well as the control cells (Figure 3, a–c). In contrast, hBMSCs treated with 10 µM Aln were less confluent than the control cells (Figure 3, d). Treatment with 1 µM Aln reduced the cell viability slightly after day 7 in group B and day 14 in group C (Figure 3, group B and group C, *p*<0.01), and 5 µM Aln was slightly cytotoxic in both groups (Figure 3, group B and group C, *p*<0.01). Treatment with 10 µM Aln caused significant cytotoxic effects after day 7 in group B and day 14 in group C (Figure 3, group B, and group C, *p*<0.01). hBMSCs treated with 1 µM Aln in group B and 1 or 5 µM Aln in group C showed similar morphologies and confluence as the control cells at the indicated days (Figure 3, e, h and i). In group B, hBMSCs treated 5 µM Aln showed only some rounded cells (Figure 3, f), but cells treated with 10 µM Aln treated cells showed cytotoxic effect and were almost completely rounded at day 15 (Figure 3, g). In group C, treatment with 10 µM Aln also induced several rounded cells at day 20 (Figure 3, j). In group D, hBMSCs treated with any dose of Aln showed no signs of cytotoxic effects during culture (Figure 3, group D), and these cells grew and adhered well as the control (Figure 3, k, l and m). In group E, the cell viability and morphology were similar to group B, and treatment 1 µM Aln slightly reduced the cell viability after day 12 (Figure 3, group E, *p*<0.01). Cells treated with 5 µM Aln showed cytotoxic effects at day 8 (Figure 3, group E, *p*<0.01) and treatment with 10 µM Aln induced significant cytotoxic effects at day 6 (Figure 3, group E, *p*<0.01). hBMSCs treated with 10 µM Aln showed an almost completely rounded morphology at day 15 (Figure 3, p), while cells treated with 5 µM Aln showed only partial rounded cells (Figure 3, o). Cells treated with 1 µM Aln were not different than the control cells (Figure 3, n). The results indicated Aln treatment during the BM incubation was slightly cytotoxic only at 10 µM (Figure 3, group A, *p*<0.01). However, hBMSCs treated with 5 µM or 10 µM Aln during the OIM incubation showed significant cytotoxicity immediately (Figure 3, group B). These results indicated that higher doses of Aln are cytotoxic during the initiation stage of osteogenesis.

### Replenishment of FPP or GGPP reversed the Aln-induced mineralization and cytotoxicity

**Figure 4:**
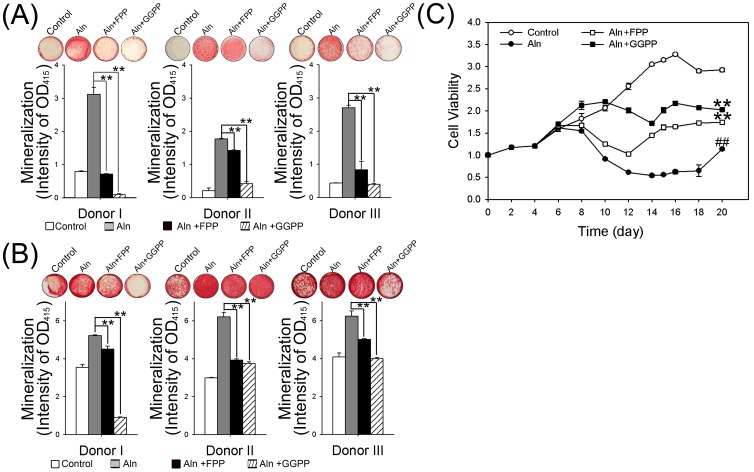
The reversal effects of FPP or GGPP on mineralization and cell viability of Aln-treated hBMSC with group B. The mineralization effects and cell viabilities of Aln-treated hBMSCs with group B were determined after additional treatment with FPP or GGPP. The ARS staining was performed at day15 (A) and day20 (B). The mineralization levels are shown as photographs and the quantified data shown as bar graph compared to 5 µM Aln-treated group. **p<0.01, n = 5, compare with 5 µM Aln treatment. (C) The cell viabilities of hBMSCs treated with control, 5 µM Aln only, 5 µM Aln with 10 µM FPP or 5 µM Aln with 10 µM GGPP were measured by MTT assays and normalized to day 0. **p<0.01, n = 4, compare with 5 µM Aln treatment; ## *p*<0.01, n = 4, compare to the control.

We measured the mineralization of hBMSCs cultures that replenished with 5 to 20 µM of FPP and GGPP. The results showed no significant difference among treatment groups (Figure S4A and S4B). Based on this data, we used 10 µM FPP and GGPP to test the mineralization of group B hBMSCs. After FPP or GGPP replenishment, the effects of Aln treatments were partially reversed. Treatment with 10 µM FPP and 5 µM Aln significantly reduced the mineralization effects observed after treatment with 5 µM Aln in all three donors. The mineralization effects decreased by approximately 78%, 20% and 69% at day 15 (Figure 4A, *p*<0.01), and 14%, 37% and 20% at day 20 (Figure 4B, *p*<0.01). Moreover, treatment with 10 µM GGPP and 5 µM Aln nearly reversed the effects observed after treatment with Aln alone (Figure 4A and 4B, decreased approximately 97%, 77% and 86% at day 15, *p*<0.01, and 83%, 40% and 36% at day 20, *p*<0.01, respectively). The alkaline phosphatase (ALP) activity assay also showed similar results. The ALP activities of FPP or GGPP replenishment at day 15 were remarked reduced than Aln alone in group B (Figure S4C). According to these results, FPP replenishment partially reversed and GGPP replenishment almost completely reversed the Aln-induced mineralization.

The MTT assay results further indicated the Aln-induced cytotoxicity was partially reversed by FPP or GGPP replenishment (Figure 4C). The viability of cells treated with 5 µM Aln was only 38.8% of control hBMSCs (Figure 4C, *p*<0.01). After co-treatment with FPP and 5 µM Aln, the cell viability increased to 59.2% of control (Figure 4C, *p*<0.01). Co-treatment with GGPP and 5 µM Aln increased the cell viability to 69.2% of the control (Figure 4C, *p*<0.01). These results suggested that the mevalonate pathway may be involved in Aln-induced osteogenesis and cytotoxicity, and FPP or GGPP replenishment not only reduced osteogenesis but also reversed Aln-associated cytotoxicity.

## Discussion

Aln was reported to enhance osteogenic differentiation in hBMSCs [9], [10]; however, the timing of Aln treatment required for osteogenic effects has not been studied. In this report, we varied the dose and treatment timing of Aln during different stages of differentiation to examine the effects of Aln on hBMSCs.

We found that the most effective treatment was 5 µM Aln administered during the initiation stage of osteo-induction; however, the higher dose of Aln inhibited cell growth and even induced cytotoxic effect on hBMSCs during the earlier stage of osteo-induction. These findings not only demonstrated the significance of timing and the optimal effective dose on enhancing hBMSC osteogenesis in hBMSCs but also demonstrated the need to avoid simultaneous cytotoxic effect. By controlling the dose and time of Aln treatment, this combined use of Aln and hBMSCs may be locally applied as a material for enhancing bone regeneration.

Previous reports describe the osteogenesis of hBMSCs in different culture medium [20]. Elevated ALP activities were observed after approximately 7 days of culture in medium either with or without dexamethasome, and this effect was maintained for two weeks. In the absence of Aln, the hBMSC osteogenesis observed in this study showed similar pattern as the previous studies. The mineralization levels increased after culturing the cells in osteo-iduction medium (OIM) for 7 to 15 days (Figure 2, day 12 to 20 in control group). In regarding the effect of Aln on differentiation, the enhanced osteogenesis observed in group A and B was significantly higher than that observed in group C and D, and this effect was most pronounced in group B. In group A, Aln treatment prior to osteo-induction, required higher dose (10 µM) of Aln to enhance osteogenesis (4.74- and 1.84-folds increases when compared to the control at day 15 and day 20, respectively). In group B, treatment with 5 µM Aln during the initiation stage of osteo-induction effectively enhanced osteogenesis, and 10 µM Aln showed an even stronger effect. In comparison to the control cells, the mineralization levels observed in group B after treatment with 1, 5 or 10 µM Aln were 1.3-, 3.9- and 7- fold higher day 15, respectively, and 1.3-, 1.5- and 2.2- fold higher at day 20, respectively. Accordingly, we suggest that Aln treatment during the initiation stage of osteogenic differentiation enhanced the osteo-induction of hBMSCs. This effect was confirmed in BMSCs obtained from 3 different donors, all of which showed similar effects (Figure 2C and 2D). Only BMSCs obtained from donor III and treated in group A showed less sensitivity to the 10 µM Aln treatment.

In previous studies, BMP-2 expression increased after 3 to 4 days of Aln treatment in osteoblastic cells [7], [8], ADSCs [11] and BMSCs [9]. However, the continuous supplement of Aln did not further increase BMP-2 expression in the later stage [10], which suggests that the initiation stage was more sensitive to the effects on Aln than the later stage. These results also suggest that Aln-induced osteogenesis in hBMSCs may act via BMP-2 expression and a subsequent increase in mineralization [10]. In this study, the continuous supplement of Aln in group E also confirmed that extending the duration of Aln treatment does not further enhance osteo-induction. The mineralization and cell viability pattern were similar to group B (Figure 2B and G; Figure 3 group B and E). Based on the findings from this study and previous studies, we suggest that the most effective timing of Aln on hBMSC osteogenesis is during the initiation stage of osteogenic differentiation.

In this study, we also investigated the cytotoxic effects of different Aln treatment strategies. In previous studies, Aln inhibited cellular proliferation at high dose in MG-63 osteoblastic cells (10^−4^ M) [7] and primary human osteoblasts (<10^−5^ M) [5]. The toxic doses of Aln were usually greater than 1 µM in differentiated cells, such as chondrosacrcoma cell [21], epidermal cell [22], endothelial cell [23], fibroblast [24], intestinal epithelial cell [25], or macrophage [3], [26], [27]. In this study, the results showed that treatment with 10 µM Aln in group A inhibited the viability of hBMSCs (Figure 3, group A), but only 5 µM Aln reduced its cell viability in groups B and C (Figure 3, group B and group C). These results revealed that the growth inhibitory or cytotoxic effect of Aln on hBMSCs might be more pronounced during the initiation stage of osteogenesis. Interestingly, the decreases of cell viability were consistent with the enhanced mineralization observed in hBMSCs treated with higher dose of Aln (Figure 2A and B; Figure 3, 10 µM in group A and 5–10 µM in group B). Accordingly, there may be some factors in Aln-induced osteo-induction that are also involved in cytotoxic effects of Aln treatment.

Recently, several studies indicated Aln suppresses bone resorption via inhibiting FPP synthase in osteoclasts, and thus suppressing osteoclastic differentiation and apoptosis [1], [2], [3], [28]. Furthermore, Aln-induced apoptosis was partially reversed by FPP or GGPP replenishment [3], [28]. However, the inhibition of isoprenylation was recently found to be involved in osteoblst osteogenesis [29], [30], [31]. The role of FPP or GGPP in the osteogenesis of osteoblast cells or human mesenchymal stem cells (hMSCs) was found to inhibit mineralization in osteoblast cultures [30], [31], [32], [33]. Furthermore, the osteoblastogenesis of hBMSCs was increased by GGPP- or FPP-specific inhibitors in Aln-treated hMSCs but no effects on cell proliferation were observed [29]. In previous study, hMSCs treated with 40 µM FPP or 10 µM GGPP alone showed no significant effect on cell proliferation and apoptosis [34]. Five to 20 µM of FPP treatments were also used in the inhibition of zoledronic acid [5] induced mineralization in osteoblasts [31], which indicated FPP (lower than 40 µM) and GGPP (lower than 10 µM) did not affect cell proliferation individually, and GGPP (higher than 5 µM) inhibited ZA-induced mineralization. In the results of BMP-2 and Runx-2 expression, also showed no significant difference between control and 10 µM FPP or GGPP treated hBMSCs (Figure S3). In this study, we found that the Aln-induced osteogenesis of hBMSC was partially reversed by FPP replenishment and completely reversed by GGPP replenishment (Figure 4A and 4B). The Aln-induced cytotoxic effects were also reversed by FPP or GGPP replenishment (Figure 4C). The results indicate that Aln-reduced cell viability were at least partially due to the inhibition of FPP or GGPP in the mevalonate pathway.

## Conclusions

We conclude that an Aln treatment strategy for hBMSC osteo-induction requires 5 µM Aln during the initiation stage of osteo-induction. A higher dose of Aln may be cytotoxic to hBMSCs at this stage. The results from this study suggest that using appropriate dose of Aln at specific time point may provide an application strategy for hBMSC-based material for tissue engineering on bone regeneration.

## Supporting Information

Figure S1 **Isolated hBMSCs were identified by surface markers and multi-potent differentiation potential.** (A) Isolated hBMSCs were identified by CD markers that measured by flowcytometry using specific antibody against CD29, CD34, CD45, CD49d, CD90 and CD166. The white diagram indicated CD marker positive population and the red diagram indicated non-specific antibody control. (B) Isolated hBMSCs were induced to osteogenesis, chondrogenesis or adipogenesis by induction medium. Alcian blue, ARS or oil red staining was for sGAG, mineralization or oil droplet, respectively.
(TIF)

Figure S2 **Aln increases osteogenic gene expressions and ALP activity in hBMSCs.** (A) The mRNA expression of BMP-2, Runx-2 and osteocalcin in 5 µM Aln-treated hBMSCs were measured at day 5 and 7. The treatment strategy was shown in Figure 1. Cultured hBMSCs were untreated (control) or treated with 5 µM in the first 5 days in bone medium (grey bars; Group A) or the second five days in osteo-induction medium (black bars; Group B). **p<0.01, n = 3; * *p*<0.05, n = 3, compared with control group. ##*p*<0.01, n = 3; #*p*<0.05, n = 3, compared with group A. (B) The ALP activities of Aln-treated hBMSCs were measured at day 15 with control group, group A, and group B.
(TIF)

Figure S3 **Treatment of FPP or GGPP did not affect the expression of BMP-2 and Runx-2 in hBMSCs.** The mRNA expression of BMP-2 and Runx-2 in FPP or GGPP-treated hBMSCs were measured through two weeks. Cultured hBMSCs were untreated (control) or treated with 10 µM FPP (grey bars) or 10 µM GGPP (black bars) in bone medium for five days and then shift to osteo-induction medium without FPP or GGPP. Compare with the control, n = 3.
(TIF)

Figure S4 **The reversal effects of different concentrations FPP or GGPP on mineralization of Aln-treated hBMSC with group B.** The mineralization effects and cell viabilities of Aln-treated hBMSCs with group B were determined after additional treatment with 5 µM (light grey bars), 10 µM (deep grey bars) and 20 µM (black bars) FPP (A) or GGPP (B). The ARS staining was performed at day 15 and day 20. The mineralization levels are shown as photographs and the quantified data shown as bar graph compared to 5 µM Aln-treated group. *p<0.05, n = 3, compare with 5 µM Aln treatment. (C) The ALP activities of 5 µM Aln-treated only or with additional 10 µM FPP or 10 µM GGPP were measured at day 15.
(TIF)

Methods S1 (DOC)
