# Supplementary figures and images for: The Susceptive Alendronate-Treatment Timing and Dosage for Osteogenesis Enhancement in Human Bone Marrow-Derived Stem Cells
Source: PLoS One. 2014 Aug 26;9(8):e105705. doi: 10.1371/journal.pone.0105705 (PMC4144913; doi:10.1371/journal.pone.0105705)

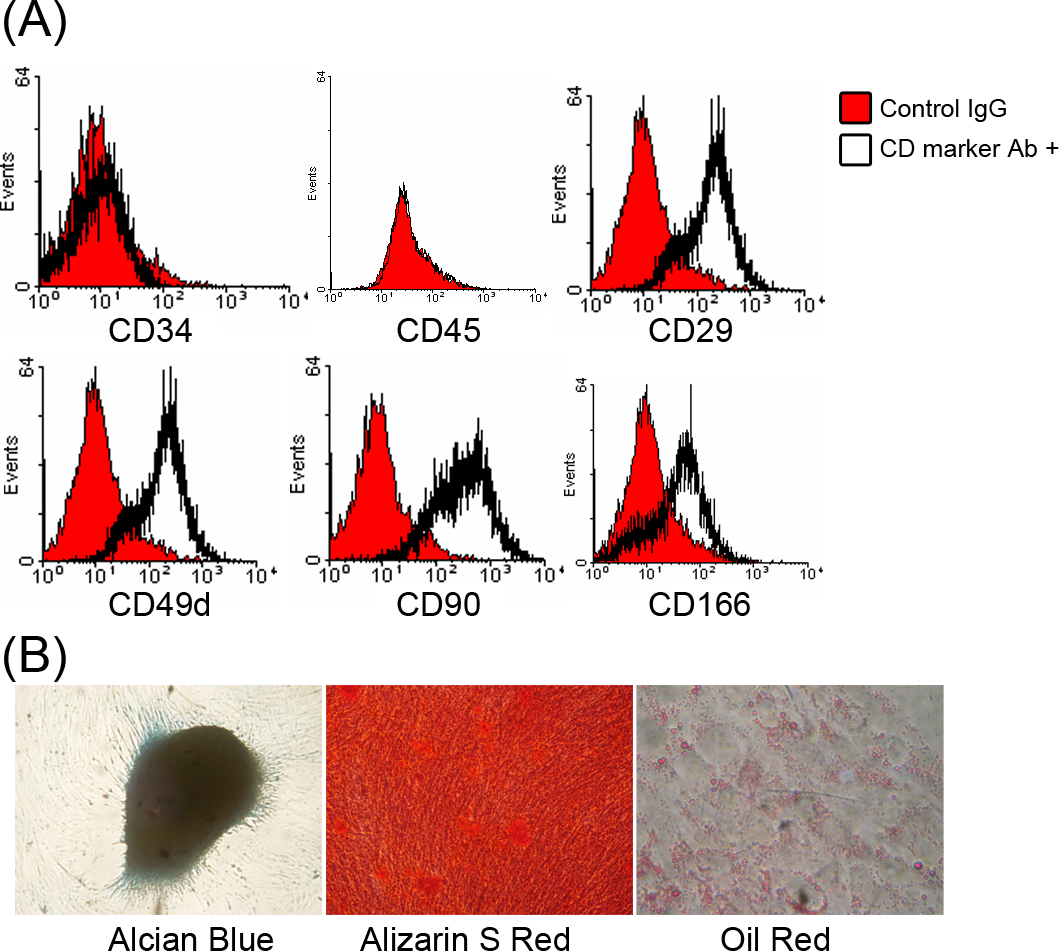

Supplement: Figure S1 — Isolated hBMSCs were identified by surface markers and multi-potent differentiation potential. (A) Isolated hBMSCs were identified by CD markers that measured by flowcytometry using specific antibody against CD29, CD34, CD45, CD49d, CD90 and CD166. The white diagram indicated CD marker positive population and the red diagram indicated non-specific antibody control. (B) Isolated hBMSCs were induced to osteogenesis, chondrogenesis or adipogenesis by induction medium. Alcian blue, ARS or oil red staining was for sGAG, mineralization or oil droplet, respectively. (TIF) [file pone.0105705.s001.tif]

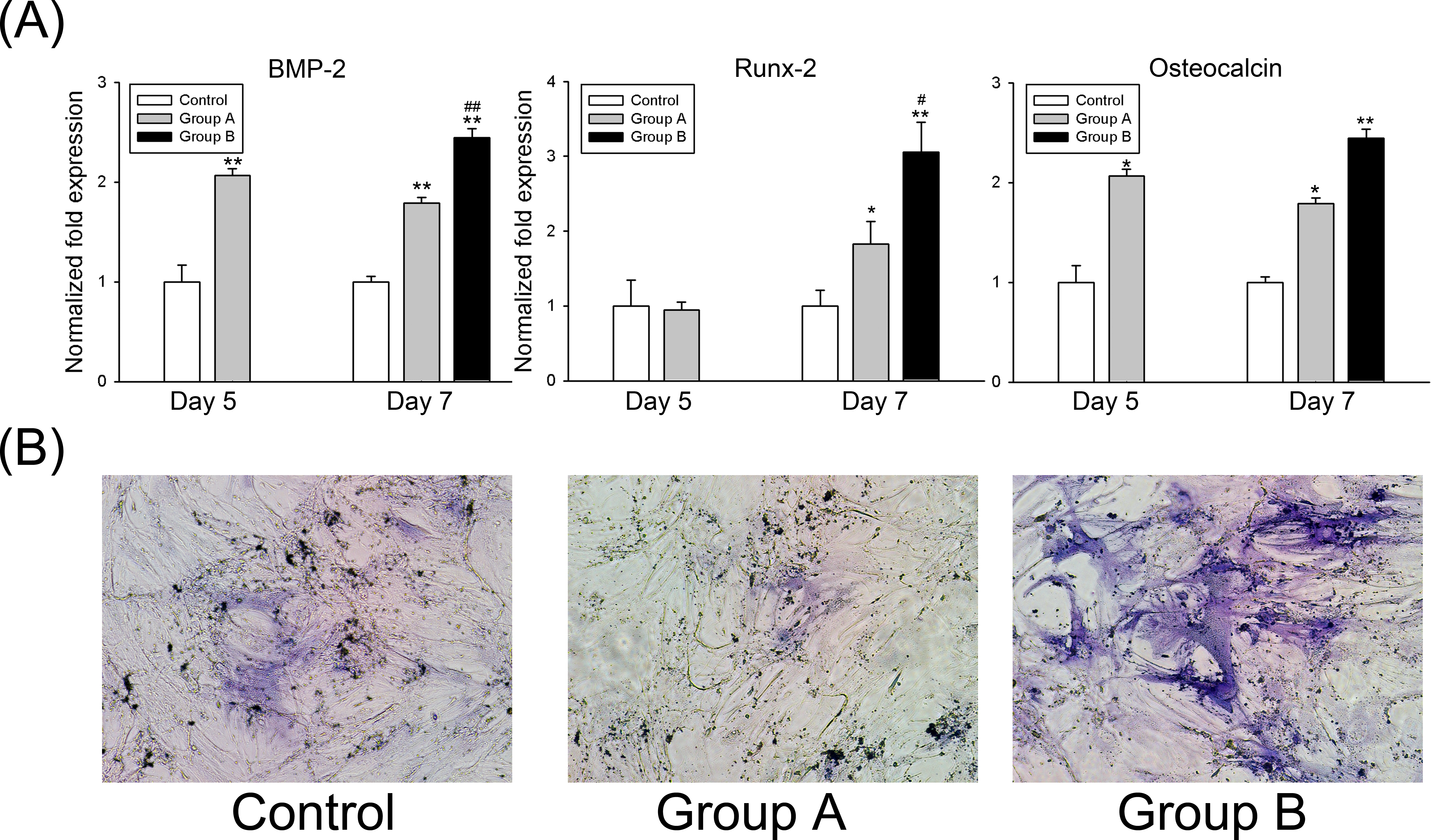

Supplement: Figure S2 — Aln increases osteogenic gene expressions and ALP activity in hBMSCs. (A) The mRNA expression of BMP-2, Runx-2 and osteocalcin in 5 µM Aln-treated hBMSCs were measured at day 5 and 7. The treatment strategy was shown in Figure 1. Cultured hBMSCs were untreated (control) or treated with 5 µM in the first 5 days in bone medium (grey bars; Group A) or the second five days in osteo-induction medium (black bars; Group B). **p<0.01, n = 3; * p<0.05, n = 3, compared with control group. ##p<0.01, n = 3; #p<0.05, n = 3, compared with group A. (B) The ALP activities of Aln-treated hBMSCs were measured at day 15 with control group, group A, and group B. (TIF) [file pone.0105705.s002.tif]

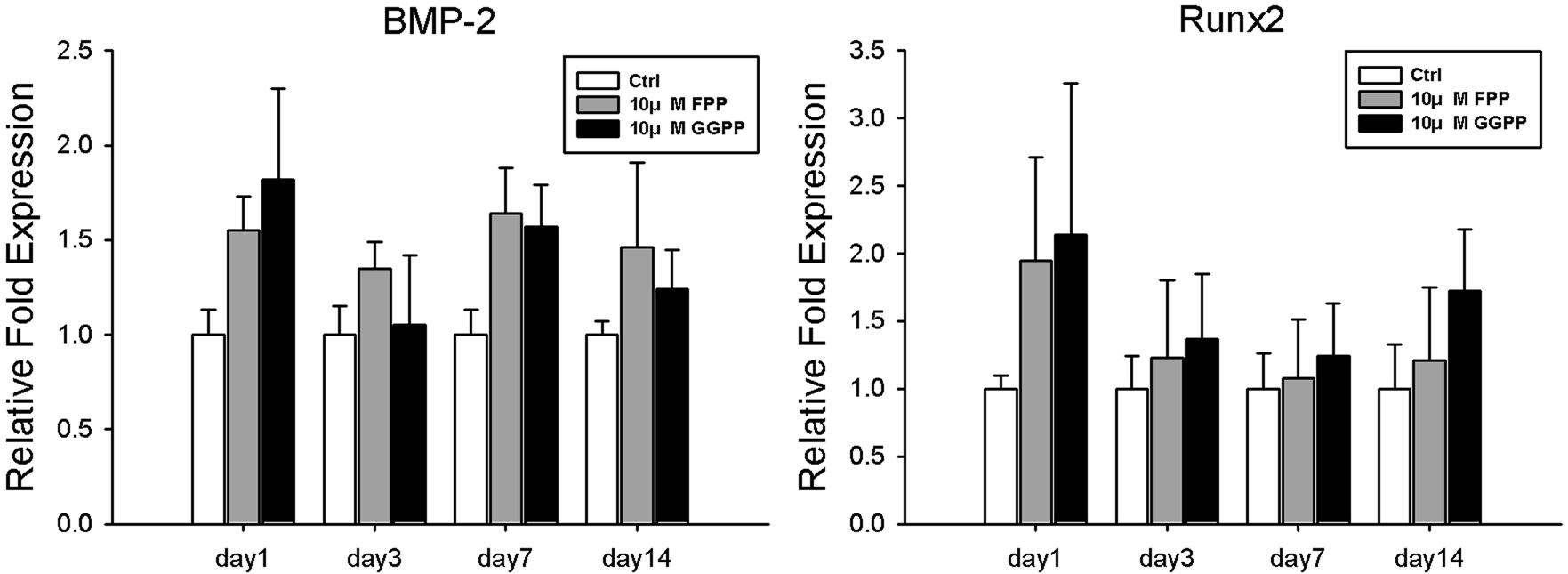

Supplement: Figure S3 — Treatment of FPP or GGPP did not affect the expression of BMP-2 and Runx-2 in hBMSCs. The mRNA expression of BMP-2 and Runx-2 in FPP or GGPP-treated hBMSCs were measured through two weeks. Cultured hBMSCs were untreated (control) or treated with 10 µM FPP (grey bars) or 10 µM GGPP (black bars) in bone medium for five days and then shift to osteo-induction medium without FPP or GGPP. Compare with the control, n = 3. (TIF) [file pone.0105705.s003.tif]

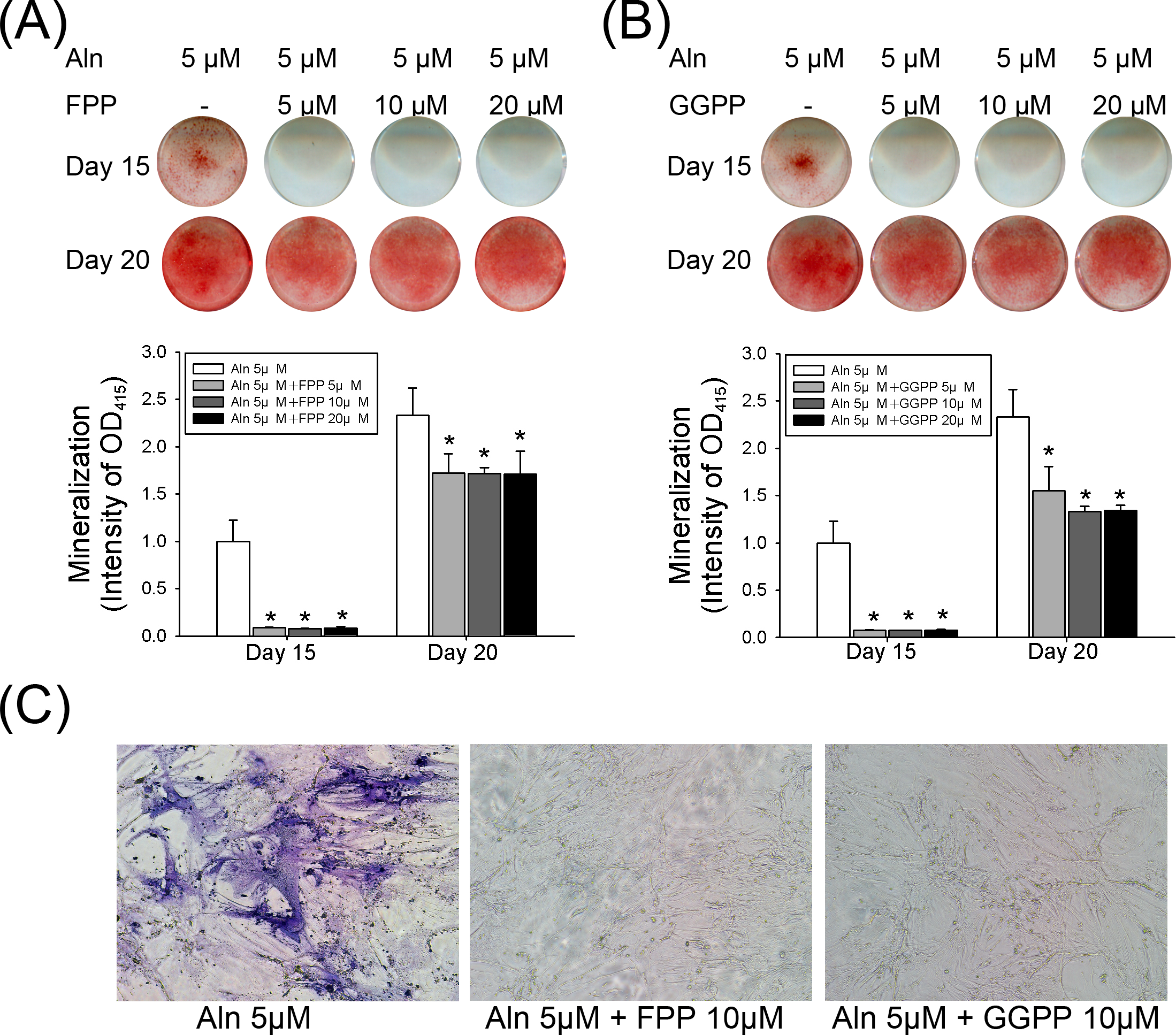

Supplement: Figure S4 — The reversal effects of different concentrations FPP or GGPP on mineralization of Aln-treated hBMSC with group B. The mineralization effects and cell viabilities of Aln-treated hBMSCs with group B were determined after additional treatment with 5 µM (light grey bars), 10 µM (deep grey bars) and 20 µM (black bars) FPP (A) or GGPP (B). The ARS staining was performed at day 15 and day 20. The mineralization levels are shown as photographs and the quantified data shown as bar graph compared to 5 µM Aln-treated group. *p<0.05, n = 3, compare with 5 µM Aln treatment. (C) The ALP activities of 5 µM Aln-treated only or with additional 10 µM FPP or 10 µM GGPP were measured at day 15. (TIF) [file pone.0105705.s004.tif]
