# Supplementary material for: The Susceptive Alendronate-Treatment Timing and Dosage for Osteogenesis Enhancement in Human Bone Marrow-Derived Stem Cells
Source: PLoS One. 2014 Aug 26;9(8):e105705. doi: 10.1371/journal.pone.0105705 (PMC4144913; doi:10.1371/journal.pone.0105705)
Supplement: Methods S1 — (DOC) [file pone.0105705.s005.doc]

**Material and Method S1**

Alkaline phosphatase (ALP) activity

Isolated hBMSCs were seeded in 24-well plates at a density of 10^4^ cells/well and followed the culture strategy shown in Fig. 1. At indicated day, cells were washed with PBS and fixed with 10% Formalin for 10 minutes. After fixation, the ALP activities were measured by alkaline phosphatase kit (Sigma-Aldrich, Saint Louis, MO, USA) followed the manual. After washed with distill water, the stained cells were observed by microscope (Eclipse TE300, Nikon, Melville, NY, USA).
